# BoNT/A1 Secondary Failure for the Treatment of Neurogenic Detrusor Overactivity: An Ex Vivo Functional Study

**DOI:** 10.3390/toxins14020077

**Published:** 2022-01-21

**Authors:** Jacquie Maignel, Vincent Martin, Rana Assaly, Mathieu L. Vogt, Kevin Retailleau, Fraser Hornby, Alexandra Laugerotte, Stéphane Lezmi, Pierre Denys, Johannes Krupp, Charles Joussain

**Affiliations:** 1 IPSEN Innovation, 91940 Les Ulis, France; vincent.martin@ipsen.com (V.M.); mathieu.vogt@orange.fr (M.L.V.); kevin.retailleau@ipsen.com (K.R.); alexandra.laugerotte@ipsen.com (A.L.); stephane.lezmi@ipsen.com (S.L.); johannes_krupp@hotmail.com (J.K.); 2 Pelvipharm, 78180 Montigny-le-Bretonneux, France; r.assaly@pelvipharm.com; 3 IPSEN Bioinnovation, Milton Park, Abingdon OX14 4RY, UK; fraser.hornby@ipsen.com; 4 Raymond-Poincaré Hospital, 92380 Garches, France; pierre.denys@aphp.fr (P.D.); charles.jsn@free.fr (C.J.)

**Keywords:** neurogenic bladder, treatment failure, botulinum A toxin, NDO, mechanism of action

## Abstract

Management of neurogenic detrusor overactivity (NDO) remains a clinical priority to improve patients’ quality of life and prevent dramatic urological complications. Intradetrusor injection of onabotulinumtoxinA (BoNT/A1, botulinum neurotoxin A1) is approved as second therapeutic line in these patients, demonstrating a good efficacy. However, a loss of its efficacy over time has been described, with no clear understanding of the underlying mechanisms. This paper aims at shedding new light on BoNT/A1 secondary failure in NDO through functional and structural analysis. Three groups of patients (either non-NDO, NDO with no toxin history or toxin secondary failure) were investigated using an ex vivo bladder strip assay. Detrusor strips were tensed in organ baths and submitted to electrical field stimulation to generate contractions. Recombinant BoNT/A1 was then added at various concentrations and contractions recorded for 4 h. Histology exploring BoNT/A1 targets, fibrosis and neuronal markers was also used. Detrusor strips from patients with BoNT/A1 secondary failure displayed a smaller sensitivity to toxin ex vivo at 3 nM compared to the other groups. Histological evaluation demonstrated the presence of cleaved Synaptosomal-Associated Protein, 25 kDa (c-SNAP25) in the detrusor from the toxin-secondary failure population, indicating some remaining in vivo sensitivity to BoNT/A1 despite the therapeutic escape. Moreover, residual c-SNAP25 did not affect parasympathetic-driven contractions observed ex vivo. This study confirms the slightly lower efficacy of BoNT/A1 in the BoNT/A1 secondary failure NDO group, suggesting that the escape from BoNT/A1 efficacy in NDO occurs at least at the parasympathetic level and could imply compensatory mechanisms for detrusor contraction.

## 1. Introduction

Worldwide, up to 80% of patients with multiple sclerosis (MS) or traumatic spinal cord injury (SCI) and more than 60% of patients with spina bifida (SB) suffer from neurogenic bladder mostly related to neurogenic detrusor overactivity (NDO) [1,2,3]. NDO induces urine leakage, significantly decreasing patients’ quality of life (QoL) leading to urological complications including urinary tract infections, urinary lithiasis and renal failure.

NDO management aims to improve patients’ QoL and to prevent urological complications by achieving regular bladder emptying without post-void residue, avoiding high intra-detrusor pressure and maintaining continence. The current management strategy is well characterized and includes intradetrusor injection of onabotulinumtoxinA (Botox^®^) as second-line treatment frequently associated with clean intermittent catheterizations [4]. OnabotulinumtoxinA was first introduced as a treatment of NDO secondary to SCI when refractory to anticholinergic treatment, by Stohrer and Schurch [5]. Since then, extensive placebo-controlled studies demonstrated efficacy and safety of this procedure, mainly in patients with SCI, MS or SB [6,7,8], allowing onabotulinumtoxinA to be supported by level 1 evidence for this indication, and approved by the Food and Drug Administration (FDA) in August 2011. The European health authorities followed with similar approval.

The main mechanism of action of botulinum neurotoxin A1 (BoNT/A1, active ingredient in onabotulinumtoxinA) in NDO is well known and relies on the cleavage of Synaptosomal-Associated Protein, 25 kDa (SNAP25). The cleavage of this membranal Soluble NSF (N-ethylmaleimide-sensitive factor) Attachment Protein Receptor (SNARE) protein inhibits neurotransmitter release by post-ganglionic parasympathetic axons, thus, decreasing detrusor overactivity [9]. It also acts at the bladder afferences level, decreasing the autocrine and paracrine action of the muscular-urothelial mechanoreceptors and the C-fibers excitation [10,11,12,13,14].

However, several studies recently demonstrated the therapeutic failure of onabotulinumtoxinA to control NDO in the long term, based on clinical and urodynamic parameters. Up to 28.9% of patients were not responsive to onabotulinumtoxinA treatment after 7 to 10 years [15,16,17]. Joussain et al. also reported the severity of NDO as a predictive factor of lack of efficacy [16]. Recently, neurogenic inflammation was put forward as an explanation for this phenomenon [18,19]. Previously, no study has compared neurogenic bladders from patients naïve to onabotulinumtoxinA therapy versus neurogenic bladders from patients with secondary failure of onabotulinumtoxinA therapy from both physiological and histological perspectives.

This paper aims to shed new light on BoNT/A1 secondary failure in NDO through a double analysis. Bladders from different populations of patients will be assessed using ex vivo pharmacology and immunohistochemistry (IHC), to try and unveil any phenomenon that may explain how patients may no longer respond to BoNT/A1 intradetrusor injections.

## 2. Results

### 2.1. Immunohistochemistry

**Figure 1:**
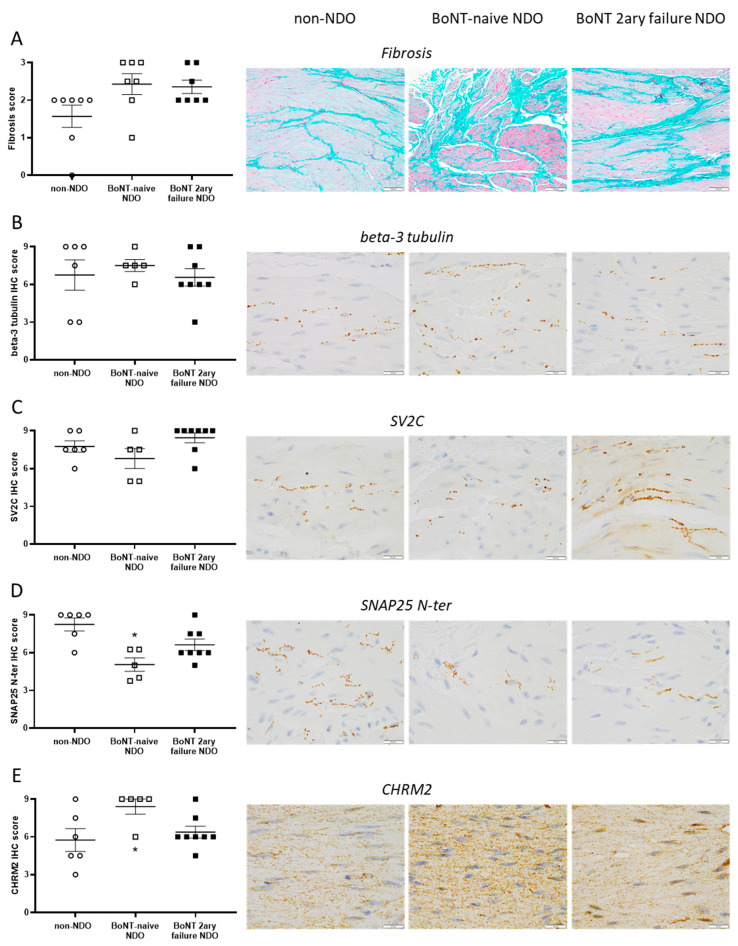
(**A**) Fibrosis score (left) and Masson’s trichrome stain (middle and right) allowing the identification of fibrosis (in green) and myocytes (in pink) in detrusor tissues from control/non-NDO, and patient with NDO naïve or with secondary failure to BoNT/A1. Scale bar is 100µm. (**B**–**E**) Staining levels (left) and immunochemistry staining (middle and right) of beta-3 tubulin (**B**), SV2C (**C**), SNAP25 N-ter (**D**) and CHRM2 (**E**) in detrusor tissues from control, and patient with NDO naïve or with secondary failure to BoNT/A1. Scale bar is 20 µm. The data are mean ± SEM of *n* = 5 to 8 detrusor sections from *n* = 5 to 8 different patients. Post-ANOVA all-pairs Tukey-Kramer or Steel-Dwass tests, * *p* < 0.05 versus control patients. All groups were compared pairwise, only significant difference was signaled, no * means NS (not significant).

Using histopathology evaluation on Masson’s trichrome slides, the severity of fibrosis in detrusor was quantified for all groups (Figure 1A). A non-significant trend for a more severe fibrosis was found for both NDO groups compared to control/non-NDO patients. No difference was noted between the two NDO populations.

The overall density of nerve endings in the detrusor was assessed using the neural pan-marker beta-3 tubulin. As seen in Figure 1B, no significant difference was found between the three populations.

The level of BoNT/A main receptor SV2C and target SNAP25 N-ter (total SNAP25) was also assessed at synapses in the detrusor of the three groups. The staining level of SV2C was found to be similar between the various populations (Figure 1C). However, SNAP25 N-ter level was significantly lower in BoNT-naïve than in control patients (Figure 1D). A similar difference was also observed when compared with secondary BoNT-failure patients but did not reach statistical significance. A trend for a lower SNAP25 level in the latter population compared with control patients was also noted.

Finally, the level of CHRM2 at the surface of myocytes in the detrusor was evaluated. NDO naïve patients showed significant higher CHRM2 levels compared with control patients, and a trend for higher levels compared with secondary BoNT-failure NDO patients (Figure 1E).

**Figure 2:**
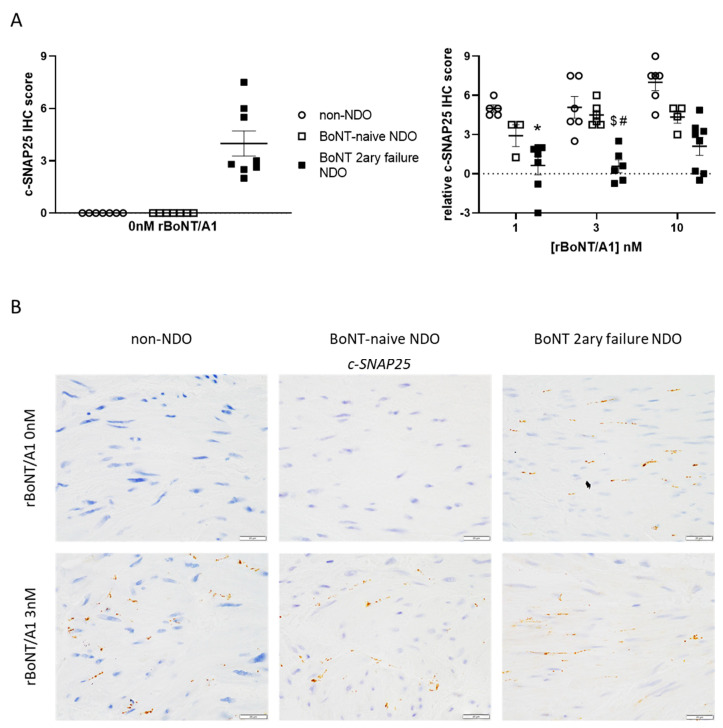
(**A**) c-SNAP25 staining levels observed in detrusor strips from control/non-NDO, and patient with NDO naïve or with secondary failure to BoNT/A1 in presence of 0 (=vehicle, left) 1, 3 or 10 nM (right) rBoNT/A1. For 1, 3 and 10 nM rBoNT/A1, the IHC score from each strip was subtracted from the paired one obtained at 0 nM. The data are mean ± SEM of *n* = 3 to 12 detrusor strips sections from *n* = 3 to 8 different patients. * *p* < 0.05 versus ‘control 1 nM’; $ *p* < 0.05 versus ‘control 3 nM’; # *p* < 0.05 versus ‘NDO BoNT-naïve 3 nM’. (**B**) Immunochemistry staining of c-SNAP25 in detrusor strips sections from control, NDO BoNT-naïve and NDO BoNT secondary failure patients submitted to 0 or 3 nM rBoNT/A1 during ex vivo assay. Scale bar is 20 µm.

The level of BoNT/A-cleaved substrate, c-SNAP25, was evaluated in the detrusor strips tested in the ex vivo assay with either buffer or rBoNT/A1 1–10 nM (Figure 2A, left). Under basal (buffer-tested) conditions, c-SNAP25 was, as expected, absent in tissues from control and BoNT-naïve NDO patients. However, a moderate staining was observed at synapses for secondary BoNT-failure population. This “residual” c-SNAP25 staining is very likely the result of intra-detrusor BoNT/A injections anterior to surgery. To assess the quantity of newly-cleaved SNAP25 by rBoNT/A1 during the ex vivo assay, c-SNAP25 IHC scores observed for each strip treated with this toxin were subtracted from paired IHC scored quantified in buffer condition. A trend for a dose-response effect was observed in control and BoNT-naïve populations, but not in secondary BoNT-failure population in which the levels of c-SNAP25 in rBoNT/A1-treated strips remained quite unchanged (Figure 2A, right). Interestingly, for rBoNT/A1 3 nM, c-SNAP25 levels were significantly higher for the control and BoNT naïve patients than the latter ones (Figure 2A, right, Figure 2B). No other significant difference was observed for the other rBoNT/A1 conditions, apart from control and secondary BoNT-failure populations at 1 nM.

### 2.2. Ex Vivo Detrusor Strip Assay

**Figure 3:**
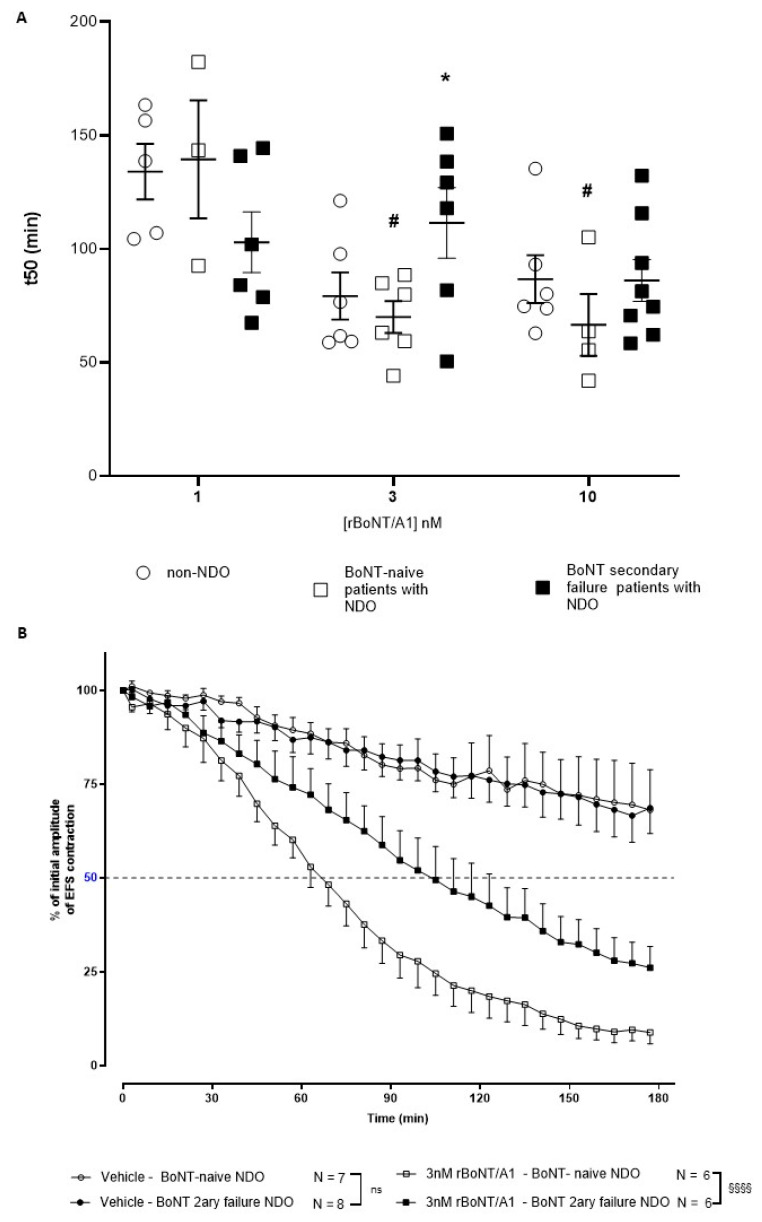
(**A**) t50s of human bladder strips from control patients (non-NDO) and patient with NDO naïve or with secondary failure to BoNT/A1 incubated ex vivo with 1, 3 or 10 nM rBoNT/A1 (*n* = 3 to 6). The data are mean ± SEM of n experiments using bladder samples from n different patients, * *p* < 0.05 versus ‘NDO BoNT naïve’ at 3 nM; # *p* < 0.05 versus ‘NDO BoNT naïve at 1 nM’. (**B**) Kinetics of force generated in the control strips and 3 nM BoNT/A1-treated for bladders from BoNT secondary failure or BoNT naïve patients with NDO (*n* = 6–8 patients/group), §§§§ *p* < 0.001.

This assay mimics smooth muscle contractions induced by the stimulated release of acetylcholine and ATP from the neural varicosities in the detrusor. It thus focuses on the implication of parasympathetic fibers in micturition. First, before the strips were subjected to EFS, the reactivity to carbachol between the three groups was similar (4.72 ± 0.35 g; 4.35 ± 0.50 and 4.63 ± 0.65 g contractions for control bladder strips, NDO naïve and NDO escaped bladder strips, respectively, mean ± s.e.m., from *n* = 7 to 8 patients). Then, rBoNT/A1 did induce a concentration-related paresis at 1, 3 and 10 nM in bladder strips from the three different populations under conditions where the vehicle did not. In detrusor strips from secondary BoNT-failure NDO patients, the t50s were not significantly different between the three concentrations of rBoNT/A1 (1, 3 and 10 nM), while in detrusor strips from botulinum toxin-naïve patients, rBoNT/A1 at 3 and 10 nM showed a significantly quicker inhibitory effect compared to 1 nM on EFS-induced contractions. Interestingly, 3 nM rBoNT/A1 induced a significantly greater and faster effect on EFS-induced contractions of strips from botulinum toxin-naïve patients compared with BoNT secondary failure patients (Figure 3A for t50s for all groups and concentrations and Figure 3B for illustration of paresis over time under vehicle or 3 nM rBoNT/A1 for NDO bladders only, in the interest of clarity). The control strips (incubated with buffer only) displayed a residual force of about 75% of the initial signal after 3 h of stimulation. There was no difference in control strip contracting behavior between naïve and BoNT secondary failure groups (Figure 3B), despite the background C-SNAP25 described by immunohistochemistry (Figure 2A).

As a result of the pre-defined exclusion criteria, several strips were excluded from analysis, explaining the range from *n* = 3 to *n* = 8 in Figure 3B.

## 3. Discussion

Here, we demonstrated that bladder strips of patients inefficiently treated by BoNT/A1 were less sensitive to the paralyzing effect of rBoNT/A1 ex vivo, with a significantly slower paresis of detrusor contractions observed at 3 nM when compared to naïve patients. Quite interestingly, in the BoNT/A1 secondary failure group, a significant amount of cleaved SNAP-25 was observed in non-treated bladder strip, despite no significant differences in the contractility over time between groups (toxin-naïve and secondary failure NDO) treated with vehicle. This point underlines the ability of the detrusor to contract despite SNAP-25 cleavage in secondary failure NDO patients. This result is consistent with previous data [20].

As the ex vivo assay mimics detrusor contractions in tissue without urothelium, these data are focusing on efferent/parasympathetic fibers innervating the bladder. This highlights that the loss of efficacy of BoNT/A1 could occur, at least partially, at the efferent level. To our best knowledge, this paper is the first to underline BoNT/A1 escapement in the detrusor itself. Moreover, SNAP25 cleavage resulting from anterior clinical onabotulinumtoxinA injections did not affect the capability of the strips from secondary failure NDO bladders to contract, suggesting an internal process overpassing the toxin effect.

From an indication point of view, pathophysiological mechanisms remain unclear and could involve remodeling at the pre-synaptic level (post-ganglionic parasympathetic nerve) or at the post-synaptic level (smooth muscle). For instance, an increase in excitatory neuromodulators release could be responsible for this escape. Indeed, it has been demonstrated that ATP and acetylcholine release could be vesicular/SNAP-25 independent [21], and thus be insensitive to SNARE-cleaving events. However, we could not investigate these elements as neither acetylcholine (ChAT or vAChT) nor purinergic (ATP or P2X3) pathways were assessed here. We may also suggest mechanisms increasing intracellular calcium to overpass BoNT/A1 SNAP-25 cleavage to achieve neurotransmitter release. Complementary experiments would be required to assess these elements. Besides, previous authors suggested that post-BoNT/A1 sprouting is very limited at the smooth muscle level compared to striated muscle [22], which was supported by our data. Thus, nerve sprouting cannot explain BoNT/A1 loss of efficacy in these patients. Additionally, some authors suggested that NDO could lead to an increased muscarinic sensitivity at the post junctional level [23,24], following a partial parasympathetic denervation, even if these conclusions remain debated [25]. However, our methodology, using bladder strips, and thus only a few square millimeters of a human bladder, could not allow us to assess accurately “patchy denervation” following BoNT/A1 injections. Unfortunately, we failed at detecting CHRM3 to interpret the involvement of muscarinic excitatory receptors. However, interestingly, CHRM2 inhibitory receptors level was increased in NDO naïve patients while SNAP-25 level was significantly lower compared to the control group. These parameters could explain the better ex vivo response of NDO-naïve patients bladder strips to BoNT/A1. We may hypothesize that the decreased presence of total SNAP-25 could be the result of a chronic retrocontrol aiming to regulate neuromediator release, while inhibitory CHRM2 receptors acting on adenyl cyclase activity, potassium channel, cation channels and TRP channels [26] would add up to this regulation. As a result, chronic BoNT/A1 injections could, despite an initial control, disturb this potential adaptation of the system and be, in the end, actors of the escapement.

Besides, natural BoNT/A1 was the protein used for the intradetrusor injections in patients, while equipotent but recombinant BoNT/A1 was used in the ex vivo assay, for technical reasons. The secondary failure is thus not specific for a product, but for a molecule and the mechanisms involved at the tissue level. However, it has been proposed that abobotulinumtoxinA (Dysport^®^, Ipsen Biopharm Ltd., Wrexham UK) could be more efficient following onabotulinumtoxinA secondary failure [27]. Based on our results we could suggest that this could more rely on the dose than on the drug itself as proposed previously [28,29].

Also, despite our results suggesting involvement of the detrusor in secondary failure, we have to consider associated phenomena, that we could not explore here. Indeed, BoNT/A1 acts at several levels in the neurogenic bladder, not only impacting the efferent pathway, but also the urothelium and the sensory neurons [12,30] and possibly the central nervous system [31]. We suppose that other pathways could be involved such as a significant increase in the afferent terminal density associated to chronic inflammation at the mucosa level in bladders, tissue fibrosis or neutralizing BoNT/A1 antibodies [18]. Further experiments remain necessary to explore these aspects.

Finally, several limitations such as the small number of patients and the pathology heterogenicity have to be considered. Moreover, groups were not totally comparable regarding age and NDO seniority. Also, we did not assess our samples’ purinergic properties or immunostaining, preventing us from exploring the involvement of ATP in the fading of BoNT/A1 efficacy, which could be important as previously explained.

## 4. Conclusions

These results underline a slightly lower efficacy of BoNT/A1 in the BoNT/A1 secondary failure NDO group in ex vivo assay despite an efficient SNAP-25 cleavage, suggesting that the escape from BoNT/A1 efficacy in NDO occurs at least at the parasympathetic level and could imply compensatory mechanisms at the detrusor level.

## 5. Materials and Methods

### 5.1. Patients Details and Medical History

A total of 15 neurogenic bladder tissue samples were obtained from augmentation cystoplasty following onabotulinumtoxinA secondary failure (BoNT/A failure) requiring surgical procedure was defined by the persistence of urinary leakage and/or urodynamic parameters (persistence of high detrusor pressures (>40 cm H_2_O) or low bladder compliance (<20 mL/cm H_2_O) [20]) in patients with NDO (*n* = 8), and non-continent urinary diversion surgery (Bricker surgery) from onabotulinumtoxinA-naïve patients with NDO proven by urodynamics (*n* = 7). All patients provided their written informed consent, including data privacy obligations. Clinical data (age, sex, therapeutics, pathology and urodynamic parameters) were extracted from patients’ clinical files according to the French legislation for retrospective studies, conforming with the reference methods (MR004 CNIL agreement number: 2209010 V0) (Table 1). Additionally, bladder tissue was obtained from patients undergoing cancer surgery (*n* = 7, ‘control bladders’). The mean age of the seven patients was 73 ± 2 years old and 100% of these patients were men.

### 5.2. Histopathology and Immunohistochemistry

The detrusor strips used in the ex vivo experiments (and thus incubated with recombinant BoNT/A1 (rBoNT/A1)) as well as remaining bladder samples from dissection were fixed in formalin, embedded in paraffin, and tissue sections (5 µm; Leica Microtome Jung RM2045, Leica Microsytems, Wetzlar, Germany) were mounted on slides. Then, sections were stained with classical Masson’s trichrome method (LAPV, Amboise, France). Severity of fibrosis was determined by a pathologist using a 4-point scale scoring system (0: absence, 1: mild, 2: marked, 3: severe). For immunohistochemical staining, a standard avidin-biotin-peroxidase procedure was used [32]. After a heat-induced epitope retrieval step, endogenous peroxidases were blocked for 10 min in 3% H_2_O_2_. Slides were incubated with primary antibodies specific for cleaved-SNAP25 (c-SNAP25) by BoNT/A (EF14007, rabbit polyclonal, IPSEN, France), all forms of SNAP25 (N-ter part) (SNAP25 N-ter) (111 011, Synaptic Systems, Göttingen, Germany), SV2C, the protein receptor for BoNT/A1 (MABN367, Merck, Darmstadt, Germany), beta-3 tubulin, a neuron-specific marker, revealing neuronal density (G712A, Promega, Fitchburg, WI, USA) or cholinergic receptor muscarinic 2, one of the muscarinic acetylcholine receptors subtypes in the bladder (CHRM2) (NLS1331, Novus Biologicals, Abingdon, UK). After incubation with a biotinylated anti-rabbit IgG secondary antibody for 30 min (Vector Laboratories, Burlingame, CA, USA), followed by a 30 min incubation with avidin-biotin coupled to horseradish peroxidase (Vector Laboratories, Burlingame, CA, USA), sections were incubated for 5 min with 0.02% diaminobenzidine (DAKO, Santa Clara, CA, USA), and counterstained with hematoxylin. c-SNAP25 was assessed in detrusor strips from ex vivo assays, while SNAP25 N-ter, SV2C, beta-3 tubulin and CHRM2 were assessed in the detrusor of the dissection bladder samples. Staining density and intensity were independently determined by a trained experimenter with a 4-point scale scoring system (0: none, 1: low density/intensity, 2: moderate density/intensity, 3: high density/intensity). More precisely, staining intensity is dependent on the amount of antigen detected, inducing more or less chromogen deposit in tissues. Density is a subjective quantification of the overall amount of positive nerves in a structure (e.g., in muscular layers). This analysis was performed by two different operators in a blind manner and verified by a board-certified veterinary pathologist (SL, ECVP). A final IHC score was calculated by multiplying the staining density score by the staining intensity score (final score from 0 to 9).

For statistical analysis, all IHC and histopathology data were analyzed using JMP Pro v15.2 (SAS Institute Inc., Cary, NC, USA, 2021). Parametric (all-pairs Tukey-Kramer) or non-parametric (Steel-Dwass) tests were used post-ANOVA. The level of significance was set at *p* < 0.05.

### 5.3. Ex Vivo Detrusor Strip Assay

All reagents were purchased from Sigma (St Louis, MO, USA) and solutions prepared on the day of the experiment. rBoNT/A1 was produced at IPSEN Bioinnovation (Milton Park, UK) in PBS/BSA and stored at −80 °C. This neurotoxin has the same primary sequence and activity equivalent to natural BoNT/A1 [32]. Tissue samples were stored at 4 °C in Krebs-HEPES buffer and transported to the research facilities immediately after surgery. The urothelium was carefully removed and eight sections of detrusor (4 × 2 × 2 mm) were excised and suspended in 5 mL organ chambers on tissue holders fitted with platinum electrodes for isometric tension recording (Pioden controls Ltd., Ashford, UK). The strips from the control group were prepared from healthy tissue, devoid of any sign of cancer infiltration or inflammation. Following the equilibration period, the detrusor strips were stimulated chemically (100 mM KCl, 3µM carbachol) and electrically (electrical field stimulation (EFS) 20 Hz, 1 ms pulse duration, 5 s train duration, 300 mA, Bionic System, Nozay, France). The viability of the strips was measured by carbachol stimulation at the beginning (CCh1) and at the end (CCh2) of the experiments. EFS trains were continuously performed by groups of 3 stimulations applied at 1-min interval and followed by a 3-min period of rest. This protocol selectively depolarizes parasympathetic fibers located in the bladder wall, as proved by the inhibition of the contractions with oxybutynin, in a previous set of experiments (internal data). Stimulations were continued until stable responses were obtained (a response was considered stable when the percentage of variation of the amplitude of EFS contractions calculated for the last three groups of EFS contraction during stabilization period was ≥90% or ≤110%). Then, individual strips were incubated with either vehicle (Krebs with 0.5% gelatin), rBoNT/A1 at 1, 3 or 10 nM, by pipetting concentrated solutions into the bath. The EFS stimulations were then continued for 3 h. The toxin-induced paresis was quantified by measuring the force of contraction over time as a percentage of the initial contraction (100%), and consequently computing the half paralysis time (t50), i.e., the time necessary to inhibit 50% of the initial contraction (Mac Lab TM/8 with Chart TM 5 software, AD Instruments Ltd., Dunedin, New Zealand). Calculations were done by fitting a four-parameter dose-response logistic curve model (Y = Bottom + (Top-Bottom)/(1 + 10^((LogIC50-X) * Hill Slope)) onto the experimental data using GraphPad Prism 8.3.0 (GraphPad Software Inc., La Jolla, CA, USA, 2021).

Exclusion criteria were defined as follows, in order to obtain a valid evaluation of the inhibitory effect of BoNT/A: the initial EFS-induced contraction force (before toxin addition) had to be higher than 300 mg, and CCh2 had to be at least 35% of CCh1.

For statistical analysis, kinetics of paresis were compared with a Bonferroni’s multiple comparison test, while differences between t50s were determined by an all pairs Tukey-Kramer’s test post one-way analysis of variance, with significance set at *p* < 0.05. All data processing and statistical tests were done in JMP Pro v15.2 (SAS Institute Inc., Cary, NC, USA, 2021).

## Figures and Tables

**Table 1:** General patient details displaying the gender, age, the presence of low bladder compliance, the neurologic disease, seniority in the disease (years), the potential treatment with antimuscarinics and the time since the last BoNT/A injections in the bladders (days). Regarding patients providing control bladders, the age ranged from 64 to 78. (MS: Multiple sclerosis, SCI: Spinal cord injury, SB: Spina-bifida, EM; Encephalomyelitis, NA: Not Applicable, Na: not available); * low bladder compliance was compliance below 20 mL/cm H_2_O, for which the individual value is given between brackets when it is the case.

Groups	Gender	Age (Year)	Low Bladder Compliance *	Neurologic Disease	Seniority of the Neurologic Disease (year)	Antimuscarinic (yes = 1; no = 0)	Number of BoNT/A Injection before Surgery	Last Intradetrusorial Injection of BoNT/A (Days)
BoNT/A-naïve patients	F	55	0	MS	21	0	NA	NA
	M	33	0	SCI	12	0	NA	NA
	F	67	0	SCI	4	0	NA	NA
	F	71	0	SCI	45	1	NA	NA
	F	52	1 (10)	SCI	38	0	NA	NA
	M	50	0	SCI	24	0	NA	NA
	M	64	0	SCI	33	0	NA	NA
BoNT/A- secondary failure patients	F	18	0	SCI	10	1	6	141
	M	32	0	SCI	10	1	5	79
	M	27	0	SCI	10	1	16	147
	F	28	1 (13)	SB	28	1	5	122
	M	31	0	SCI	8	1	6	37
	F	60	Na	EM	14	1	14	256
	M	21	Na	SCI	8	1	5	412
	M	37	1 (10)	SCI	5	1	4	163

## Data Availability

Data sharing not applicable.
